# FPGA-Based Pedestrian Detection for Collision Prediction System

**DOI:** 10.3390/s22124421

**Published:** 2022-06-11

**Authors:** Lucas Cambuim, Edna Barros

**Affiliations:** Centro de Informática, Universidade Federal de Pernambuco—UFPE, Recife 50740-560, Brazil; ensb@cin.ufpe.br

**Keywords:** pedestrian detection, high performance, distant pedestrian, image pyramid, multi-window, histogram of oriented gradients, support vector machine, collision prediction efficiency

## Abstract

Pedestrian detection (PD) systems capable of locating pedestrians over large distances and locating them faster are needed in Pedestrian Collision Prediction (PCP) systems to increase the decision-making distance. This paper proposes a performance-optimized FPGA implementation of a HOG-SVM-based PD system with support for image pyramids and detection windows of different sizes to locate near and far pedestrians. This work proposes a hardware architecture that can process one pixel per clock cycle by exploring data and temporal parallelism using techniques such as pipeline and spatial division of data between parallel processing units. The proposed architecture for the PD module was validated in FPGA and integrated with the stereo semi-global matching (SGM) module, also prototyped in FPGA. Processing two windows of different dimensions permitted a reduction in miss rate of at least 6% compared to a uniquely sized window detector. The performances achieved by the PD system and the PCP system in HD resolution were 100 and 66.2 frames per second (FPS), respectively. The performance improvement achieved by the PCP system with the addition of our PD module permitted an increase in decision-making distance of 3.3 m compared to a PCP system that processes at 30 FPS.

## 1. Introduction

Pedestrians, called vulnerable road users (VRUs), represent more than half of all the global deaths in transit accidents [1]. As cars today get faster, the risk of fatal accidents involving pedestrians increases even further [1]. Pedestrian collision prediction (PCP) systems are fundamental in reducing accidents because they allow earlier decision-making [2].

Pedestrian detection (PD) is a fundamental and critical component in PCP systems [3,4]. Since braking distance increases with larger vehicle speed [5], two aspects are essential in these PD systems and should be applied in PCP systems to support higher speeds: (1) coverage in large ranges of distances and (2) fast response. PD systems with such characteristics permit PCP systems to predict a collision earlier [6].

One way to work around the deficiency of detecting distant pedestrians is to process data with increasing resolutions [7]. Image sensors (cameras) are an attractive choice because they provide a large amount of environmental information in pixels. This technology is constantly evolving [8,9] and, today, it is possible to have high-resolution cameras with high sampling rates [10,11,12]. Higher resolution cameras facilitate the detection task and allow the system to detect distant pedestrians, as they will be encoded in a greater number of pixels. For example, in [13] the authors claim that increasing the resolution from 480 × 320 to 1920 × 1080 pixels allows for increasing the detection distance by 77 m. In addition, a higher frame rate improves obstacle tracking, reduces latency, and minimizes the false detection rate [13].

Unfortunately, the existing detectors based on convolutional neural networks (CNN) have a high computational cost that prevents us from obtaining efficient processing solutions [14,15]. For example, in [14] the authors process images in resolution 416×416 at 60 FPS. On the other hand, solutions that combine a histogram of oriented gradients (HOG) with shallow linear classifiers such as support vector machines (SVMs) can achieve high processing rates [13] due to their relatively regular and straightforward processing.

Due to the excellent parallelism capabilities of the field-programmable gate array (FPGA) platforms [16,17,18], some architectures for HOG-SVM-based detectors have been proposed. The proposed detectors in [13,19] do not support the image pyramid, which is one of the fundamental approaches to locating pedestrians nearer to the image. They propose approximations to avoid using the arc-tangent and so as to not perform the linear interpolation in the cell computation that reduces detection accuracy [20].

In [21] the authors propose a detection system with support for pyramids based on a heterogeneous architecture that is heavily dependent on external memory performance. The HOG and SVM steps are processed in FPGA, and the other steps are performed in general-purpose processors (GPP), such as color conversion, gradient, and the image pyramid. Only one HOG and SVM module is used to process all the pyramid levels iteratively. Thus, it is impossible to continuously process all the frames without input pixel interruption.

In [22] the authors propose a detection system with support for the image pyramid entirely in FPGA that effectively processes only six scales per frame. The authors do not perform parallelism to process the considerable overlapping along an image row. Consequently, the authors used a double frequency strategy to increase the SVM module’s processing throughput, which is non-scalable in terms of frequency and increases hardware complexity.

In addition to the image pyramid, another approach to increasing the ability to locate pedestrians at different distances involves processing detection windows of different dimensions. While the pyramid aims to encounter pedestrians larger than the detection window dimension, the windows of various sizes permit locating pedestrians that appear with smaller dimensions. Our previous work [6] demonstrated that the combination of detectors and the image pyramid enabled finding pedestrians further and further away. However, the processing rate of the GPU-based solution proved to be non-scalable in terms of image resolution.

Thus, this work proposes an FPGA-based processing architecture of our detector [6] to further enhance the PCP system processing performance. The proposed detector is similar to the works [13,19,22], but does not approximate the cell calculation nor adopt a double frequency strategy. In addition to the pyramid, the detector supports processing windows of different sizes. The entire detection system is performed on FPGA hardware and without external memories. The detection architecture supports streaming pixels that could come straight from a hardware camera without the need for specific heterogeneous architectures.

The proposed architecture comprises several parallelism strategies at the frame level and detection windows level to performance optimization. At the frame level, modules are instantiated to process detection windows of equal and different dimensions parallel at each pyramid level. At the detection windows level, the most significant contribution of this work, a set of smaller SVM units is created to process a bunch of different overlapping detection windows in parallel to ensure the completion of the current row before the arrival of the next row. These strategies guarantee a processing throughput of one pixel per cycle, achieving the same performance as the input camera without frame loss or pixel input interruption.

In addition to the PD system architecture, another essential contribution to the performance improvement of the PCP system is in the pedestrian distance estimation step that depends on the results of the Semi-Global Matching (SGM) [23]. Since the SGM demands a high computational cost, our high-performance FPGA-based SGM [24] was integrated into the PCP system along with the proposed detector.

A heterogeneous platform based on a general-purpose processor (GPP) and FPGA implements the proposed PCP system. The GPP processes the less computationally expensive steps. The adopted platform is the Intel HARP version 2 (HARPv2) [25]. The impact of the proposed PD system is evaluated based on the pedestrian location, which is essential information in the PCP system. The image pyramid strategy with windows of different dimensions permitted a reduction of at least 6% in the miss rate compared to detectors with a detection window of a single size. The PD system processing rate for HD images (i.e., 1280 × 720) is approximately 100 FPS. The proposed detector can take advantage of event-based sensing approaches and pixel-parallel CMOS image sensors to achieve even higher processing rates and reduce power dissipation and hardware resources [11,12,26].

The impact of the proposed PD system on the collision prediction task is also evaluated. Synthetic collision scenarios were created involving an occluded pedestrian crossing in front of the moving car to carry out this assessment. The processing rate of the entire PCP system for processing HD images is approximately 66.2 FPS. This rate allowed an increase of 3.3 m in decision-making distance for a vehicle traveling at 60km/h compared to our previous PCP system [6] which processes the collision prediction at 30 FPS.

In summary, the contributions of this work are pointed out as follows:

- A highly parallel architecture with performance optimization of the HOG-SVM-based detector with support for detection windows of different dimensions and the image pyramid.
- A strategy involving several SVM units to parallel processing of a massive amount of detection windows.
- Performance optimization of the PCP system by the integration of the proposed detector and the SGM module.

This paper is organized as follows: Section 2 describes the techniques used in each step of the PD system. Section 3 describes the new proposed PD architecture. Section 4 describes the complete collision prediction system adopted, heterogeneous architecture, and HW/SW communication strategy. Section 5 presents an evaluation of the precision and performance of the proposed hardware architecture in pedestrian localization and collision prediction. Finally, conclusions are drawn in Section 6.

## 2. Pedestrian Detection (PD) System

This section describes the techniques and algorithms of the pedestrian detection (PD) system. It is essential to make it clear that this section is not the contribution of this work but of our previous work [6]. It serves to support the description of our proposed hardware architecture in Section 3.

**Figure 1:**
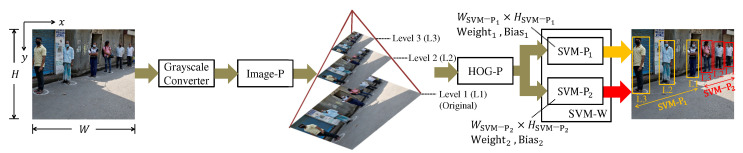
The general architecture of the PD system. The terms *W*, *H*, image-P, HOG-P, and SVM-P mean width, height, Image pyramid, HOG pyramid, and SVM pyramid.

The (PD) system steps are shown in Figure 1. The PD system aims to find pedestrians and highlight them by bounding boxes which are the extreme points $\left(x_{1},y_{1}\right)$ and $\left(x_{2},y_{2}\right)$ of a rectangle.

The detector starts converting each pixel from the RGB to the grayscale color space by weighted sum per channel. The image pyramid step (also called Image-P) produces several re-scaled images of smaller dimensions than the original image. At level 1 of the pyramid is the original image, while at the other levels, the images are re-scaled through resizing. The scale factor $S_{\mathrm{pyr}}$ defines the dimension ratio from one level to the next, and the number of pyramid levels is determined by the depth $D_{\mathrm{pyr}}$.

The HOG and SVM pyramid steps (they are called HOG-P and SVM-P) apply the HOG and SVM techniques to each image generated by the image pyramid. Each ${\mathrm{SVM}-P}_{k}$ processes detection windows of a different size $W_{{\mathrm{SVM}-P}_{k}}\times H_{{\mathrm{SVM}-P}_{k}}$. The set of SVM−Ps makes up the SVM−W, and the amount of SVM−Ps is defined by $Q_{\mathrm{SVM}-P}$.

**Figure 2:**
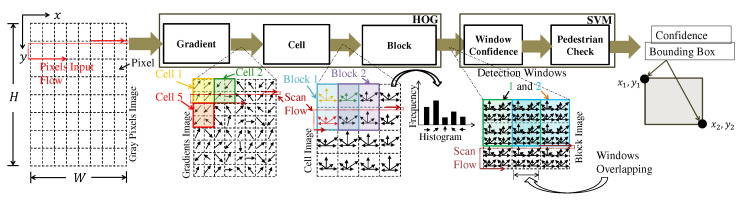
HOG and SVM overview. The SVM scans the block image using detection windows and determines whether or not each window contains a pedestrian. Colors help to differentiate cells, blocks, and detection windows.

The HOG and SVM processing steps are shown in Figure 2. HOG calculates features image based on pixel gradients. The features are histogram bins calculated by the cell and block steps. The SVM is responsible for performing the window scan on the HOG features and determining if each window (it is called detection window) contains a pedestrian or not. Briefly, in each detection window, the SVM applies the dot product between the HOG features vector and the SVM weights vector and checks if the result is above a threshold to affirm that this window contains a pedestrian. As shown in Figure 2, a detection window is formed by several HOG blocks. Different SVM-Ps have different weights and biases obtained in the supervised learning phase.

Experimentally, it was possible to observe that the HOG-SVM based detectors can only detect pedestrians whose image dimensions fit close to the limits of the detection window dimension. The image pyramid is one of the approaches to adjusting the dimension of larger pedestrians. Pedestrians that are much smaller than the detection window are not detectable due to the property of the HOG and SVM combination that cannot capture pedestrians in high pose variability. Although the samples of various sizes are added in the learning phase, the SVM classifier does not converge to a model that captures all these variabilities.

On the other hand, smaller detection windows permit to detection of smaller pedestrians that are usually farther away from the vehicle because these pedestrians fit better on these windows. The image pyramid helps increase detection capability, but the increase in the pyramid depth above a limit provokes the detector to introduce too many false positives, besides not being able to find larger pedestrians. This is because smaller detection windows contain less edge information needed to classify pedestrians.

The solution proposed in [6] is to have specialized classifiers in a certain range of distances. In the example of Figure 1, the classifiers in ${\mathrm{SVM}-P}_{1}$ have a window dimension greater than in ${\mathrm{SVM}-P}_{2}$. Thus, ${\mathrm{SVM}-P}_{2}$ can find pedestrians in a distance range more distant than ${\mathrm{SVM}-P}_{1}$.

Another aspect of this system is the data processing flow at each step. The execution can follow any flow at the software level once the data is in memory. However, on hardware systems, the execution follows the left-to-right and top-to-bottom streaming flow similar to pixel scanning of traditional hardware cameras.

The resize, HOG, and SVM steps are detailed in the following sections.

### 2.1. Resize

This step resizes the grayscale source image to a target dimension using the bilinear interpolation technique [27]. This technique calculates the grayscale intensity $I_{\mathrm{dst}}$ of each pixel in the resized image by summing the weighted grayscale intensity of four closest pixels around a given calculated position $\left(x_{\mathrm{src}},y_{\mathrm{src}}\right)$ in the source image. This position is calculated using the Equation (1).

(1) $$\begin{array}{c} x_{\mathrm{src}}=\left(\left(x_{\mathrm{dst}}+0.5\right)\cdot S_{\mathrm{pyr},x}-0.5\right)\\ y_{\mathrm{src}}=\left(\left(y_{\mathrm{dst}}+0.5\right)\cdot S_{\mathrm{pyr},y}-0.5\right) \end{array}$$

The constants $S_{\mathrm{pyr},x}$ and $S_{\mathrm{pyr},y}$ are the ratios of input width by output width and input height by output height. These constants equal $S_{\mathrm{pyr}}$, meaning that the reduction ratio in the two dimensions is the same. The terms src and dst mean, respectively, source and destination. From the position $\left(x_{\mathrm{src}},y_{\mathrm{src}}\right)$ the value of the four weights of neighboring pixels is calculated: left-weight, right-weight, top-weight, and bottom-weight. They are described, respectively, by Equation (2) as $\mu_{l}$, $\mu_{r}$, $\mu_{t}$, and $\mu_{b}$.

(2) $$\begin{array}{c} \mu_{r}=\left(x_{\mathrm{src}}-\left\lfloor x_{\mathrm{src}}\right\rfloor \right)\\ \mu_{l}=1.0-\mu_{r}\\ \mu_{b}=\left(y_{\mathrm{src}}-\left\lfloor y_{\mathrm{src}}\right\rfloor \right)\\ \mu_{t}=1.0-\mu_{b} \end{array}$$

Finally, the intensity $I_{\mathrm{dst}}\left(x_{\mathrm{dst}},y_{\mathrm{dst}}\right)$ of the target pixel located at $\left(x_{\mathrm{dst}},y_{\mathrm{dst}}\right)$ is obtained from Equation (3).

(3) $$\begin{array}{cc} I_{\mathrm{dst}}\left(x_{\mathrm{dst}},y_{\mathrm{dst}}\right)= & \;I_{\mathrm{src}}\left(\left\lfloor x_{\mathrm{src}}\right\rfloor ,\left\lfloor y_{\mathrm{src}}\right\rfloor \right)\cdot \mu_{l}\cdot \mu_{t}\;+\\ \; & \;I_{\mathrm{src}}\left(\left\lfloor x_{\mathrm{src}}\right\rfloor +1,\left\lfloor y_{\mathrm{src}}\right\rfloor \right)\cdot \mu_{r}\cdot \mu_{t}\;+\\ \; & \;I_{\mathrm{src}}\left(\left\lfloor x_{\mathrm{src}}\right\rfloor ,\left\lfloor y_{\mathrm{src}}\right\rfloor +1\right)\cdot \mu_{l}\cdot \mu_{b}\;+\\ \; & \;I_{\mathrm{src}}\left(\left\lfloor x_{\mathrm{src}}\right\rfloor +1,\left\lfloor y_{\mathrm{src}}\right\rfloor +1\right)\cdot \mu_{r}\cdot \mu_{b} \end{array}$$

The resized image is obtained by calculating all pixels within the new dimensions of the rescaled image, using Equations (1)–(3).

### 2.2. HOG

In this step, the necessary features are calculated for the classification. The HOG method comprises three main steps: gradient, cell, and block.

#### 2.2.1. Gradient

This step calculates the magnitude and the orientation angle of the gradient of each pixel from the input image. By using Dalal’s formulation in [20], the 1-D spatial gradient components $d_{x}$ and $d_{y}$ are firstly calculated according to Equation (4).

(4) $$\begin{array}{c} d_{x}\left(x,y\right)=I_{\mathrm{gray}}\left(x+1,y\right)-I_{\mathrm{gray}}\left(x-1,y\right)\\ d_{y}\left(x,y\right)=I_{\mathrm{gray}}\left(x,y+1\right)-I_{\mathrm{gray}}\left(x,y-1\right) \end{array}$$

From the pair $d_{x}$ and $d_{y}$, the magnitude m(x,y) and orientation θ(x,y) are computed through Equation (5).

(5) $$\begin{array}{c} m\left(x,y\right)=\sqrt{d_{x}{\left(x,y\right)}^{2}+d_{y}{\left(x,y\right)}^{2}}\\ \theta \left(x,y\right)=\arctan\frac{d_{y}\left(x,y\right)}{d_{x}\left(x,y\right)} \end{array}$$

The gradient image is obtained by calculating the gradients of all the pixels using Equations (4) and 5, as shown in Figure 2.

#### 2.2.2. Cell

**Figure 3:**
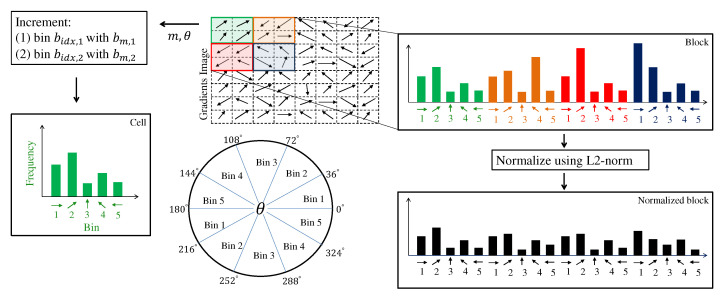
Demonstration example of HOG cell and block calculation. The colors of each histogram are to refer to its respective cell. The number below each bar is to indicate the bin index in the histogram. In this example, $Q_{\mathrm{bins}}$ is equal to 5.

In this step, the entire image formed by gradient modules and orientations is divided into grids of dimensions $W_{\mathrm{cell}}\times H_{\mathrm{cell}}$ pixels in which each rectangle is called cells, as shown in Figure 2. In each cell, a histogram is calculated. The number of bins is defined by $Q_{\mathrm{bins}}$. These bins represent well-spaced angles in $[0^{\circ }$, $180^{\circ }]$. Figure 3 demonstrates the cell and block calculation.

The magnitudes and angle of each gradient within the boundaries of a given cell define which bins will accumulate in this cell. The orientations are unsigned, meaning that angles in $[180^{\circ }$, $360^{\circ }]$ are the same as $[0^{\circ }$, $180^{\circ }]$. In mathematical terms, the unsigned orientation angle $\theta_{u}$ of a given gradient of location (x,y) is given by:

(6) $$\theta_{u}\left(x,y\right)=\left\{\begin{array}{cc} \theta (x,y), & \mathrm{if}\;0^{\circ }\leq \theta \left(x,y\right)<180^{\circ }\\ \theta (x,y)+\pi , & 180^{\circ }\leq \theta \left(x,y\right)<360^{\circ } \end{array}\right.$$

The values of the two neighboring bins are accumulated by weighted magnitude values based on the difference between the angle $\theta_{u}$ and the main bin. To obtain these weighted magnitude values, first the index $b_{idx,1}$ is calculated through Equation (7) that locates the main bin.

(7) $$\begin{array}{c} b_{\mathrm{idx},1}\left(\theta_{u}\right)=\left\lfloor \theta_{u}\cdot \frac{Q_{\mathrm{bins}}}{\pi }\right\rfloor \end{array}$$

The calculation of the difference between the angle $\theta_{u}$ and the main bin, denoted by α, is given by the following Equation:

(8) $$\begin{array}{c} \alpha \left(\theta_{u}\right)=\left(\theta_{u}-b_{\theta_{u}}\left(b_{\mathrm{idx},1}\left(\theta_{u}\right)\right)\right)\cdot \frac{Q_{\mathrm{bins}}}{\pi }, \end{array}$$

where $b_{\theta_{u}}$(i) is a function described by Equation (9) that defines the angle of a given bin index $i\in [0,Q_{bins}-1]$.

(9) $$\begin{array}{c} b_{\theta_{u}}\left(i\right)=i\cdot \frac{\pi }{Q_{\mathrm{bins}}} \end{array}$$

From $\alpha (\theta_{u})$, the portions of the gradient module that will go to the main bin ($b_{m,1}$) and the next neighboring bin ($b_{m,2}$) are calculated through Equations (10) and (11), respectively.

(10) $$\begin{array}{c} b_{m,1}\left(\theta_{u},m\right)=\left(1.0-\alpha \left(\theta_{u}\right)\right)\cdot m \end{array}$$

(11) $$\begin{array}{c} b_{m,2}\left(\theta_{u},m\right)=\alpha \left(\theta_{u}\right)\cdot m \end{array}$$

It is important to highlight that the neighbor bin index $b_{\mathrm{idx},2}\left(\theta_{u}\right)$ is calculated as:

(12) $$b_{\mathrm{idx},2}\left(\theta \right)=\left\{\begin{array}{cc} b_{\mathrm{idx},1}\left(\theta_{u}\right)+1, & \mathrm{if}\;b_{\mathrm{idx},1}\left(\theta_{u}\right)<Q_{\mathrm{bins}}\\ 0, & \mathrm{otherwise} \end{array}\right.$$

The Equation (12) guarantees that if the main bin chosen is the last one in the histogram, its neighbor bin will be the first.

#### 2.2.3. Block

The cell histograms are grouped into blocks and normalized within the block to increase immunity to lighting variations, as shown in Figure 2 and Figure 3. The block dimension is given by $W_{\mathrm{block}}\times H_{\mathrm{block}}$ pixels. The normalization step uses the Euclidean norm, called L2-norm, defined as:

(13) $$\begin{array}{c} p=\frac{v}{\sqrt{{∥v∥}^{2}+\epsilon_{L2}}}, \end{array}$$

where $p=\{p_{1},\ldots ,p_{q},\ldots ,p_{s}\}$ is the normalized block vector, $v=\{v_{1},\ldots ,v_{q},\ldots ,v_{s}\}$ is the non-normalized one-dimensional vector formed by the concatenation of the cells bins within the block, as shown in Figure 3, and $\epsilon_{L2}=0.01$ is some small constant to avoid zero division. Since blocks are made up of cells, the block’s dimension in pixels must be a multiple of the cell dimension. Therefore, the block dimension can also be expressed as cell units. All normalized vectors form the HOG features. For more details concerning the HOG algorithm, refer to [20].

### 2.3. SVM

This step performs the scan using sliding window techniques on the HOG features image. In each detection window, the existence, or lack thereof, of a pedestrian is checked. Detection windows of a given ${\mathrm{SVM}-P}_{h}$ have dimensions $W_{{\mathrm{SVM}-P}_{h}}\times H_{{\mathrm{SVM}-P}_{h}}$ pixels. The scan stride parameter $P_{{\mathrm{SVM}-P}_{h}}=\left(P_{{\mathrm{SVM}-P}_{h},x},P_{{\mathrm{SVM}-P}_{h},y}\right)$ defines the jump between one detection window and the next in the directions *x* and *y*, respectively. This parameter must be a multiple of the HOG cell dimension.

In each detection window, the descriptors are formed by concatenating the HOG features into a single one-dimensional vector. Then, the system verifies the existence of a pedestrian through the SVM linear classifier that compares these descriptors with a reference model produced by a supervised learning phase. Since it is a binary classifier, SVM linear will return the result whether or not the window contains a pedestrian. Therefore, it is crucial to guarantee that the HOG features composition order in the one-dimensional vector in the test phase is the same as in the SVM training phase. Otherwise, the classification will not work correctly.

In mathematical terms, a detection window is indicated by $u=\{u_{1},\ldots ,u_{r},\ldots ,u_{d}\}$, where each $u_{r}$ is a HOG feature, that is, a bin. The classification of this window is performed by the linear SVM function described by the following Equation:

(14) $$\begin{array}{c} f\left(u\right)=w^{T}\cdot u+b \end{array}$$

**Figure 4:**
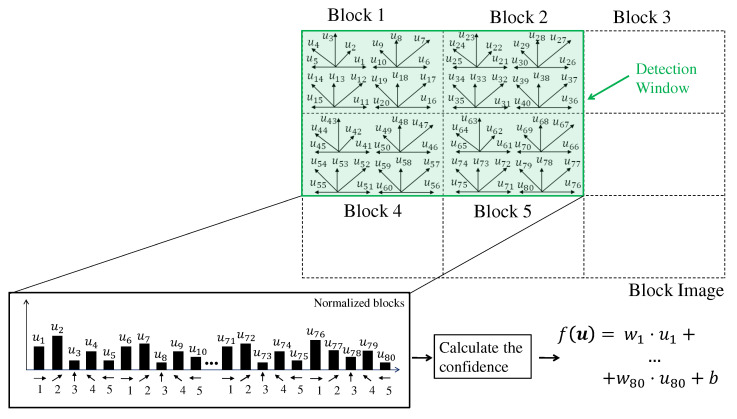
Example of the SVM function used inside a detection window. Each feature *u* corresponds to the frequency of its respective bin in the block histogram.

The parameters w and *b* are, respectively, the weight and bias of the linear SVM, defined through the supervised learning phase. The result f(u) indicates a distance value from the input u to the hyperplane separating the two classes. The higher this value, the greater the certainty in informing that the detection window contains a pedestrian. Figure 4 shows an example of using the linear SVM function over a detection window in the block image.

The function $g(u,\sigma_{SVM})$ described by Equation (15) is used to define the class of classifier response.

(15) $$g\left(u,\sigma_{\mathrm{SVM}}\right)=\left\{\begin{array}{cc} 1, & \mathrm{if}\;f\left(u\right)>\sigma_{\mathrm{SVM}}\\ -1, & \mathrm{otherwise} \end{array}\right.$$

The constant $\sigma_{\mathrm{SVM}}$ defines the confidence score. For each detection window in which the detector stated that it contains a pedestrian, its bounding box is returned.

## 3. The Proposed PD System Hardware Architecture

**Figure 5:**
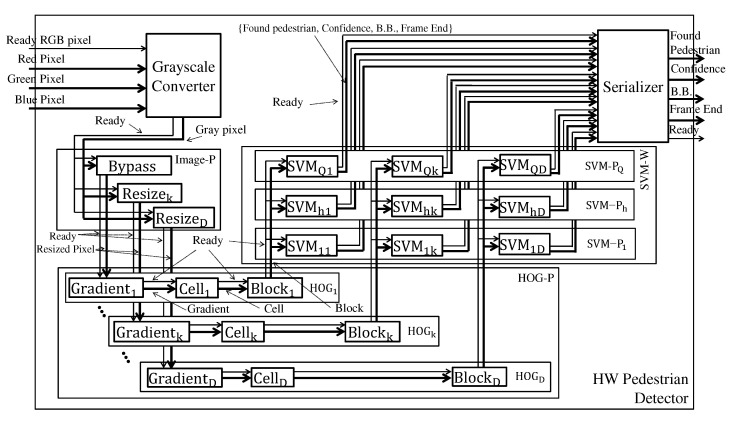
The general PD system hardware architecture. The terms *Q* and *D* is a simplification for the terms $Q_{\mathrm{SVM}-P}$ and $D_{\mathrm{pyr}}$ respectively. The term B.B. means bounding box.

This section describes the hardware architecture of the PD system, detailing the modules that implement each step of the system described in Section 2. Figure 5 shows the general hardware architecture of the proposed PD system. To facilitate the comprehension, the module names have been carefully defined to match the step names in Figure 1 and Figure 2.

A fully parallel processing strategy is required to support image pyramid and different size detection windows strategy without frame loss. The Image-P, HOG-P, and SVM-P modules instantiate, respectively, resize, HOG, and SVM modules to process each image in parallel according to the $D_{\mathrm{pyr}}$ depth and pyramid scale $S_{\mathrm{pyr}}$ parameters. The first instantiates $D_{\mathrm{pyr}}-1$ Resizes (in level 0 there is no resize, but instead a Bypass module), and the second and third instantiate $D_{\mathrm{pyr}}$ HOGs, and $D_{\mathrm{pyr}}$ SVMs modules. The scale parameter $S_{\mathrm{pyr}}$ defines the image resolution that each instantiated module will work with.

The system receives pixels in the RGB color space and returns the bounding boxes (B.B.) along with their respective confidence. The signal Found Pedestrian has value 1 when the detection window is classified as having a pedestrian (i.e., obey the Equation (15)), and 0 otherwise. The signal Ready informs when there is an output ready. The signal Frame End informs that the processing of the whole image was concluded.

All modules follow the pixel scan flow of most hardware cameras, from left to right and top to bottom. Furthermore, the PD system works in a slave mode, always waiting for the external source to inform that it has a pixel available. The PD system supports one pixel per clock cycle. Whenever the available signal is at logic level 1, an input pixel is available, and the PD system readily reads the three channels of that pixel and processes them. This slave approach is only possible because all modules can operate without interrupting the input flow of pixels. This feature allows the PD system to easily integrate through digital pins in hardware cameras that use a serial protocol [28].

In addition, the entire system operates as a continuous flow of processing. Whenever any module has data ready in the input, it will process it and store the partial results. If any response data can already be provided, the module will signal for the next module to receive this data and process it.

All modules of this system operate with numbers in fixed-point representation. All arithmetic operations come from integer operators adapted for fixed-point. For simplicity, bits wide of the fractional part, defined by $F_{\mathrm{frac}}$, are equals for all modules. The bits wide of the integer part depend on each module’s range of possible values. Without loss of generalization, the details of the bits wide of the integer part are not presented.

All modules process data in the pipeline with a minimum of operations per stage. Any mathematical operation module (i.e., multiplication, division, square root) will always have pipeline cycles quantity equal to the operated data width. Consequently, the modules have more pipeline stages, but on the other hand, the PD system achieves higher processing frequencies. After filling the pipeline stages with data, the system processes one detection window per clock cycle with no frame loss.

Next, the hardware modules that implement each step in the proposed PD system are detailed.

### 3.1. Grayscale Converter

An RGB pixel has three channels, in which the intensity of each channel has $S_{p}$ bits wide, which, in general, is equal to 8. Once a pixel is available, it is converted to grayscale by three fixed-point multipliers that multiply each channel by a respective weighting constant. Next, a two-level sum tree scheme is employed to sum the partial multiplication results. Finally, the gray pixel of $S_{p}=8$ bits wide is obtained by truncating the result from the tree of sums.

### 3.2. Resize

The resize module will process the new image converted to grayscale. The module has as parameters the resolutions of the input image and the output image defined from the $S_{\mathrm{pyr}}$ parameter and the respective level at the moment of the pyramid instance.

The processing follows the Equations (1)–(3). The first two Equations are responsible for mapping positions between the resized image and the source image, and the third is responsible for actually processing the bilinear interpolation.

Two critical aspects can be noted in these Equations. The first aspect is that if $S_{\mathrm{pyr}}$ is less than 2, the mappings from adjacent positions in the resized image will point to the same position in the original image. In other words, it is necessary to process two adjacent pixels in the resized image for the same pixel in the original image. If the two pixels are not processed in parallel, some of them will be lost. The second aspect concerns the operations that need to be performed to process Equations (1) and (2). These Equations require arithmetic operations that demand many clock cycles, leading to the input pixels lost when performing these operations.

**Figure 6:**
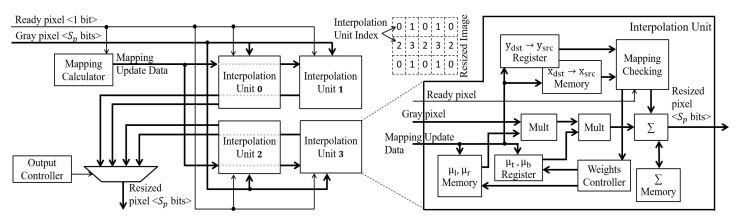
The resize architecture. The term Mult means Multiplier.

The first aspect is solved by implementing a strategy of multiple interpolation units. These units process Equation (3) at interleaved positions in the resized image, as shown in Figure 6. Units 0 and 2 process even columns, and units 1 and 3 process odd columns of pixels in the resized image. Units 0 and 1 process even rows of pixels, and units 2 and 3 process odd rows of pixels. When there are pixels in the input image that need to be used to calculate pixels at adjacent positions in the resized image, these four interpolation units will process these adjacent pixels in parallel.

Each interpolation unit has a Mapping Checking module that verifies which input pixels will participate in calculating the new resized pixels. Consider the calculation of a given pixel *q*. When the input image pixel referring to the first calculation of *q* is available, it is multiplied by the weights $\mu_{l}$, $\mu_{t}$ and stored in a register, following Equation (3). When the pixel in the next column is available, it is multiplied by $\mu_{r}$, $\mu_{t}$, added to the previous result, and stored in FIFO (First-In-First-Out) memory. This memory stores a row of partial results. When the pixel of the following line, which is part of the same *q*, is available (i.e., third pixel), it is multiplied by $\mu_{l}$ and $\mu_{b}$, added to the result of the accumulation of the previous line, and stored in a register. When the last pixel is available, it is multiplied by $\mu_{r}$ and $\mu_{b}$, added to the result of the register and made available in the output of the interpolation unit.

The second aspect is solved by noting that the scale $S_{\mathrm{pyr}}$ is constant throughout the execution. Thus, the mappings are always the same from one image to another. Therefore, a strategy in the resize module is implemented to calculate the mappings and weights and store them in memory before the resize module starts processing the new image.

The resize does not store this mapping and weight data for the entire image, as it would not be a scalable solution concerning image resolution. Instead, the Mapping Calculator module defines a whole line of mappings $x_{\mathrm{dst}}\to x_{\mathrm{src}}$ following Equation (1), split, and stores them in the FPGA memory of each interpolation unit. This is possible once the mapping on the *x* axis does not vary.

The mapping $y_{\mathrm{dst}}\to y_{\mathrm{src}}$ is defined throughout the processing. The Mapping Calculator sets the next mapping whenever the row is processed. Once the units process rows in interleaved positions, the next mapping is defined for two rows ahead. Consequently, the interleaved approach guarantees enough time for the unit to get the next mapping before the input pixels are available.

### 3.3. HOG

The HOG module includes three submodules following the steps detailed in Section 2.2: Gradient, Cell, and Block.

#### 3.3.1. Gradient

As the resized pixels are ready, this module first calculates the gradient components $d_{x}\left(x,y\right)$ and $d_{y}\left(x,y\right)$ described by Equation (4). This calculation uses the sliding window technique with a kernel of 3×3. The kernels for $d_{x}$ and $d_{y}$ are defined by Equation (16). The sliding window includes row buffers that keep track of three rows of the input image. It permits access to the target pixel’s neighborhood, which is necessary to calculate the gradient kernel.

(16) $$d_{x}=\begin{array}{ccc} 0 & 0 & 0\\ -1 & 0 & 1\\ 0 & 0 & 0 \end{array}\;\;\;d_{y}=\begin{array}{ccc} 0 & -1 & 0\\ 0 & 0 & 0\\ 0 & 1 & 0 \end{array}$$

As the components $d_{x}\left(x,y\right)$ and $d_{y}\left(x,y\right)$ are available, the modules m(x,y) and orientations θ(x,y) are calculated following Equation (5). The first component is calculated by multiplying each component $d_{x}$ and $d_{y}$ by itself to generate $d_{x}^{2}$ and $d_{y}^{2}$. Then the results are summed to generate $d_{x}^{2}+d_{y}^{2}$. Finally, its square root is calculated.

The second component θ(x,y) applies the division operation to generate $d_{y}/d_{x}$ and then the arctan. The arctan module based on coordinate rotation digital computer (CORDIC) demands many processing resources and limits the maximum frequency of operation [29]. Thus, an arctan module based on a look-up table is implemented. This module stores the arctan function mappings in FPGA memory and is fully pipelined and parameterizable in terms of fixed-point precision and extremes of the arctan. In addition, the module includes pipeline delay stages to align module and orientation results temporally.

#### 3.3.2. Cell

**Figure 7:**
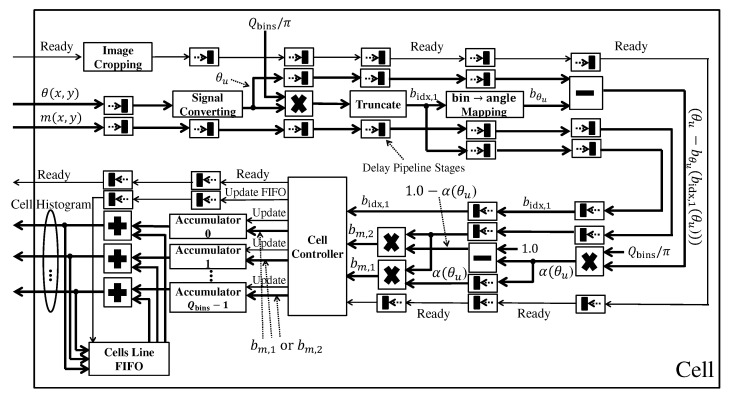
The cell histogram architecture.

As the gradient module and orientation are available, the cell module calculates the cell histograms. The processing architecture is shown in Figure 7. Firstly, the next step performs cropping of the original image, removing some rows and columns to the right and below to ensure that the cropped image is a multiple of the cell dimension. This cropping checks if the input pixel is within the cropped image boundaries in hardware. If yes, then the pixel will be outputted. The next step converts signed to unsigned orientations ($\theta_{u}$), as defined by Equation (6), performing the sum of the constant π for the angles (θ) between $[180^{\circ }$,$360^{\circ }]$.

Following Equation (7), the unsigned orientation is multiplied by the constant $Q_{\mathrm{bins}}/\pi$. The integer part of this result is extracted by the truncate module to get the main bin $b_{\mathrm{idx},1}$. This value is used in the bin→angle mapping module to searches for the respective angle $b_{\theta_{u}}$, as described by Equation (9). Instead of performing a multiplication, this mapping module defines an indexable array of constant values, in which each position $i\in [0,Q_{\mathrm{bins}}-1]$ is equal to $i\cdot (\pi /Q_{\mathrm{bins}})$.

Then, as part of the α calculation, $\theta_{u}-b_{\theta_{u}}\left(b_{\mathrm{idx},1}\left(\theta_{u}\right)\right)$ is calculated and the result is multiplied with $Q_{\mathrm{bins}}/\pi$. From α, it is obtained 1.0−α which is the weight of the neighboring bin. Then, the value of the proportional gradient modules for the two bins, $b_{m,1}$ and $b_{m,2}$, are calculated through two multiplications.

From the value of the gradient index $b_{\mathrm{idx},1}$, the Cell Controller module defines which accumulators will have their values accumulated simultaneously with the proportional gradient modules. When a row of a given cell is processed, the Cell Controller stores the accumulators’ temporary results. These values are summed with temporary values from already processed previous rows of the respective cell. The FIFO memory stores temporary results of all cells that were processed up to the previous row of the image. The sum results are stored back in this FIFO. The result no longer needs to be held back in the FIFO by processing the last row of a given cell. After finishing the processing of this row, the Cell Controller module signals that the cell is ready and its histogram is available in the output.

#### 3.3.3. Block

**Figure 8:**
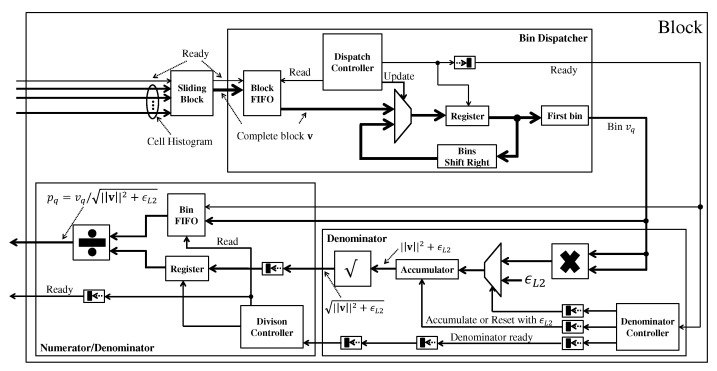
The block normalization architecture.

From the result of each cell, the block processing module performs the normalization of each bin within a block through Equation (13). The architecture of this module is shown in Figure 8. The first module performs the sliding window strategy to obtain the four cells that make up a given block in each clock cycle. Then, this block is sent to the Bin Dispatcher module that dispatches one bin at a time. Firstly, this module stores in FIFO all the blocks that come. Then the Dispatch Controller reads a block from this FIFO and stores it in the register. At each clock cycle, the first bin from the register is outputted, and the register data is shifted to discard this first bin. When all bins have been dispatched, the Dispatch Controller reads the next block from the FIFO.

Each bin goes to the Denominator module, which calculates the denominator from Equation (13). First, the power of 2 of each bin is calculated through the multiplication module and then accumulated. When all bins in the block are accumulated, their square root is calculated. This result composes the denominator. This value is sent to the Numerator/Denominator module, which calculates the normalized bin. First, the denominator is stored in a register, and the Division Controller module dispatches one bin at each clock cycle to be divided by the denominator, and then the result is outputted.

It is important to note that the one-bin-at-a-time processing approach is only possible if the number of cycles required to process a line of blocks is less than the number of cycles waiting for the following line of blocks. Otherwise, some strategy to lock reading or buffering would be necessary. This feature exists because, between one block row and the next, there are $H_{\mathrm{cell}}$ rows of cells to be formed, and this is the free space that the block module has to calculate all the blocks of the current row.

### 3.4. SVM

As the blocks (that is, features) are available, the SVM module calculates the confidence f(u) of each detection window following the Equation (14) and then checks if each window contains pedestrians, following Equation (15).

Overlapping between windows is a challenging aspect of achieving streaming processing. Since the stride in pixels between neighboring windows is smaller than the detection window dimension, multiple windows will overlap, as shown in Figure 2. Consequently, when a HOG feature is ready, it needs to be processed by several windows. To be more precise, the maximum amount of different overlapping windows $O_{\mathrm{windows}}$ that is processed from a given feature is given as:

(17) $$\begin{array}{c} O_{\mathrm{windows}}=\frac{W_{{\mathrm{SVM}-P}_{h}}}{W_{\mathrm{cell}}}\cdot \frac{H_{{\mathrm{SVM}-P}_{h}}}{H_{\mathrm{cell}}} \end{array}$$

Due to overlapping, whenever a HOG feature is ready, the SVM module will spend the number of cycles defined by Equation (17) times the number of cycles to process a unique window. It is not difficult to see that, without any parallelism, the input throughput of features will be much higher than the throughput of processing all windows. This difference in throughput certainly makes the streaming processing of the detection system unfeasible.

SVM units are proposed to process a subset of detection windows in parallel to deal with this problem. These units are arranged in $N_{r}$ rows and $N_{c}$ columns. The detection windows in the *x* direction are divided equally among the $N_{c}$ SVM units. The units SVM arranged in rows calculate detection windows of the following rows. The start detection windows line $y_{0}$ of an SVM unit that is located on line *r* in the SVM units matrix is $y_{0}=r$.

**Figure 9:**
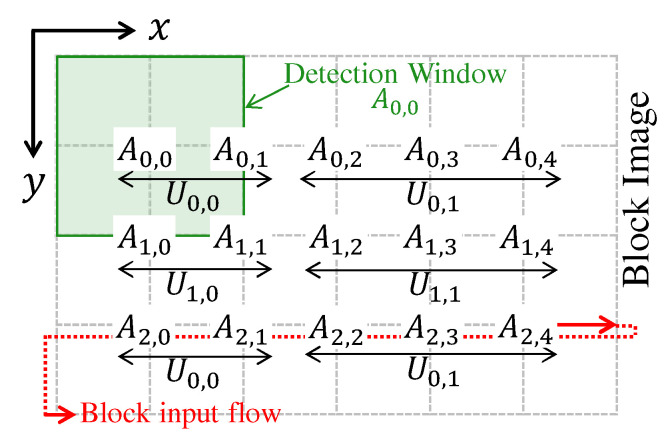
SVM Units Distribution Example. In this example, $N_{c}=2$, $N_{r}=2$, and the detection window dimension is 2 × 2 blocks. The terms $U_{r,c}$ and $A_{y,x}$ mean respectively SVM unit and detection window.

An example of this distribution of detection windows by SVM units is shown in Figure 9. In this example, there are two SVM units in the directions x (i.e., $N_{c}=2$) and y (i.e., $N_{r}=2$), and the detection window dimension is 2 × 2 blocks. As can be seen, the unit $U_{0,0}$ processes detection windows $A_{0,0}$ and $A_{0,1}$, the unit $U_{0,1}$ processes detection windows $A_{0,2}$, $A_{0,3}$, and $A_{0,4}$, the unit $U_{1,0}$ processes detection windows $A_{1,0}$ and $A_{1,1}$, and the unit $U_{1,1}$ processes detection windows $A_{1,2}$, $A_{1,3}$, and $A_{1,4}$. As can be noted, when the number of windows cannot be equal for each SVM unit of a given line, the SVM units of the last column have more windows.

Whenever any SVM unit ends all detection windows, it jumps to process a row that is not yet processed by any other SVM unit. The next line to be processed is the sum of the current line *y* of a given SVM unit and the $N_{r}$. In the example of Figure 9, it can be seen that in addition to the windows $A_{0,0}$ and $A_{0,1}$, the SVM unit $U_{0,0}$ processes the windows $A_{2,0}$ and $A_{2,1}$. If the new line exceeds the limits of the block image, then the position reverts to the initial value *r*.

The $N_{c}$ parameter value has to be defined to guarantee the completion of the current line set of blocks before the block’s arrival on the following line. This requirement ensures uninterrupted processing flow. Since the SVM units are reused to process detection windows of the following rows, the $N_{r}$ parameter value only needs to equal the number of block lines of the detection window.

**Figure 10:**
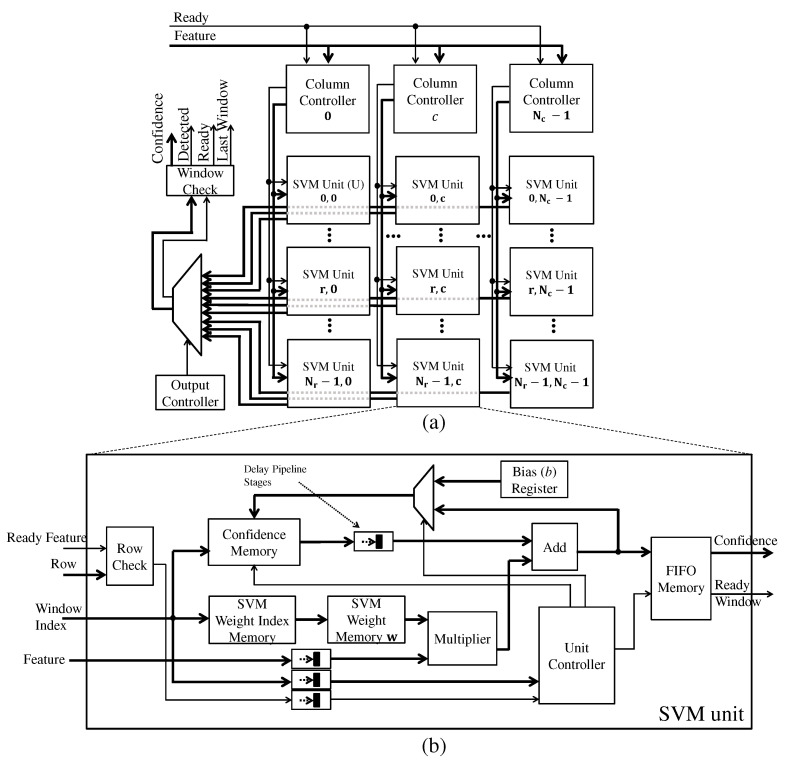
The SVM architecture: (**a**) Units matrix and management (**b**) SVM unit.

The architecture that processes this processing logic is shown in Figure 10. Column controllers temporarily store the HOG features (i.e., blocks) of detection windows in FIFO memory for each *c* column. Whenever a feature is available in this FIFO, the column controller sends it to all SVM units of a given column *c*. The controller also sends the indices of detection windows that belong to this feature. Since the processing follows a sequential and pipeline strategy within the SVM unit, the same feature is sent several times in each clock cycle with the window index value different to cover all the windows that need to be processed. When all indexes are sent, that feature is finished, and the column controller fetches the next available feature in the FIFO.

The detection window processing through the SVM unit starts by accessing the SVM weight index from the detection window index. The SVM weight value, obtained from the weight index, is multiplied by the input feature. This value is added to the temporary confidence value stored in an indexable memory and stored back in this memory. This memory stores the temporary confidence values of all detection windows.

When the last feature of a given detection window is processed, the confidence f(u) is ready. At this point, the weight index is zeroed to process a new window in another row. Furthermore, the confidence value stored in memory returns to the initial value, which is the bias *b* from Equation (14).

Neighboring SVM units can finish some detection windows simultaneously or out of order. Since the reading of results is done one at a time and in order, this concurrence of SVM units can lead to the loss of results. To ensure that the SVM unit results are not lost, they are stored in a small output FIFO as shown in Figure 10b.

The Row Check module checks if the input features belong to SVM units of their respective row. For example, features from line 0 do not belong to SVM units of the line 1, as shown in Figure 9. When it belongs, this module emits a signal to the Unit Controller to update the confidence memory and index memory with the new temporary confidence value and the next SVM weight index.

The Output Controller module in Figure 10a manages the outputs of all SVM units. This module ensures that the reading and delivery of results from the SVM units are in order. For example, if the following SVM unit (to be read) has results stored in the FIFO, the output controller sets the switch so that data from that SVM unit are read and delivered to the Check Window module. This module, in turn, checks whether or not the window contains a pedestrian following Equation (15) and also checks whether it is the last window in the image.

### 3.5. Serializer

The Serializer module manages the detection results of SVM modules from all SVM-Ps modules in a single output. This module has a set of FIFO memories, one for each SVM module, and writes its detection results from windows classified and the end-of-frame information to this FIFO. The Window Check module from the SVM module signals the processing of the last detection window of an image. A signal from an external source is required for the Serializer module to start reading FIFO data of the current frame. The Serializer reads these results one at a time from the FIFOs and outputs them. The Serializer looks for the following FIFO when obtaining the end-of-frame information. When finishing the processing of the last FIFO, the Serializer informs the ending of a frame processing, goes back to the beginning, and waits for the external signal to read the FIFOs with the next frame’s data.

## 4. Case Study: The PCP System Heterogeneous Architecture

The PD system was integrated into our PCP system [6]. Section 4.1 describes the steps and strategies used in the PCP system, and Section 4.2 describes the hardware/software integration strategy.

### 4.1. PCP System

**Figure 11:**
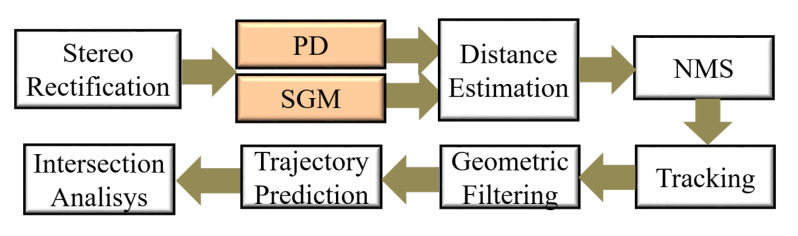
The PCP system general architecture.

Figure 11 shows the PCP system that has a stereo camera and inertial sensors attached to the vehicle to capture stereo frame pairs and vehicle movement data such as speed and yaw rate. The Stereo Rectification step performs radial and tangential distortions corrections and horizontal alignment of each frame pair.

The Stereo Matching step calculates disparity maps that permit us to estimate the pedestrian’s distances. In this step, our FPGA-based module [24] that processes the Semi-Global Matching (SGM) technique [30] in streaming and without frame loss is adopted. This technique propagates throughout the image, matching cost information through four one-dimensional paths producing more robust disparity maps for the urban context. Besides, this module supports the detection of mismatched disparities that are important to reduce distance measurement errors.

The Distance Estimation step calculates each pedestrian’s footpoint’s lateral and longitudinal distances from the bounding boxes and disparity map. Finally, the Non-Maximum Suppression (NMS) step removes several neighboring detections from the same pedestrian.

The Tracking step identifies and labels the footpoints that belong to the same pedestrian over several consecutive frames. This step uses an extended Kalman filter (EKF) with vehicle motion compensation for tracking to model pedestrian motion. In addition, the tracking also includes the Hungarian method for the global association and the gate method to exclude unlikely associations.

The Geometric Filtering step removes detections that did not meet the pedestrian locality and shape restrictions. Next, the Trajectory Prediction step estimates the future trajectories of the pedestrians and vehicles in several discrete steps. Finally, the Collision Analysis step identifies possible collision positions when pedestrians’ positions are at the same time in the future touching the vehicle. The techniques and parameters used in each of these steps are detailed in [6].

### 4.2. HW/SW Integration

**Figure 12:**
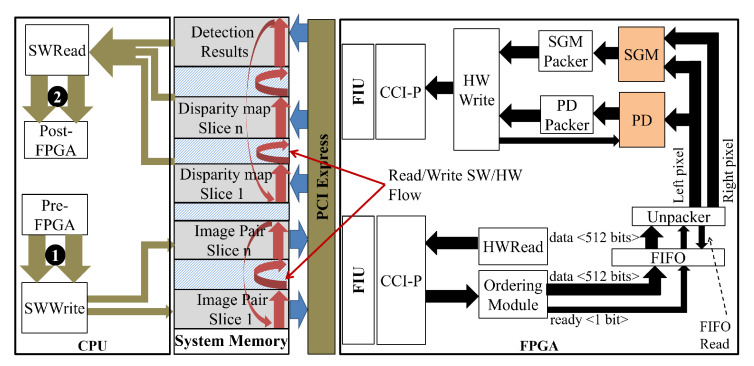
The PCP System heterogeneous architecture. The arrow labeled with the number 1 means the left and right rectified images. The arrow labeled with the number 2 means the disparity map and detection results. Pre-FPGA stands for the steps prior to FPGA processing (i.e., rectification). Post-FPGA stands for the steps after the FPGA processing (i.e., distance estimation, NMS, and so on).

Figure 12 shows the integration of the PD and SGM system in the PCP system. The Intel HARP version 2 platform (HARPv2) [25] was adopted, which consists of an Intel Xeon E5-2699 v4 CPU and a Programmable Acceleration Card (PAC) with Intel Arria 10 GX FPGA. This platform has a framework called Open Programmable Acceleration Engine (OPAE) that permits the software running in the processor to communicate with FPGAs by allocating shared memory regions in the CPU. FPGAs have access to send or send receive data.

Using OPAE, the SWWrite function writes each new pair of stereo frames in the unidimensional shared array putting each pair of stereo pixels together. On the hardware side, the HWRead module requests these pixels through the CCI protocol (CCI-P) [31] in burst mode. Solutions to support hardware/software sharing of high-resolution images are presented in our work [32].

Each data packet that arrives from the CCI-P is 512-bits wide and can hold more than a pair of pixels. The packages are sorted and stored in a FIFO so that the Unpacker module reads a pair of pixels at a time (RGB pixels of the left and right image). The left image pixel is sent to the proposed PD module, and the pair is sent to the Matching Stereo (SGM) module. Both modules run in parallel. As the results are provided from these modules, they are packed into 512-bit data by the SGM Packer and PD Packer modules.

As the memory write is performed one packet at a time, it is necessary that the writing of the results of one module ends so that the results of the next module are written. The HWWrite module does this management and sending of packets to memory. First, it sends the disparity map data. When it finishes sending, it asks the PD module to send the detection results stored in the Serializer module as described in Section 3.5.

To send or receive several frames, both the software and the hardware implement a synchronization mechanism based on exchanging parameters and control variables. Every time the software finishes writing all the data in memory, it modifies a control variable. When the HWread module recognizes the data available, it requests the reading of these data. Likewise, when the SGM and PD module finishes processing and writing all results in memory, the HWWrite module modifies the respective control variables so that the CPU knows that all results are ready and available for further processing. More communication details can be found in our work [32].

## 5. Results

The proposed PD system hardware architecture has been implemented in SystemVerilog language and validated through timing simulations using the ModelSim 10.5b and Quartus 17.1 Standard for synthesis tools. The proposed HW-based PD system (HW PD system) is evaluated in the case study: the PCP system. Section 5.1 evaluates the accuracy of the pedestrian location component. Section 5.2 evaluates the processing performance of the HW PD and PCP systems. Then, Section 5.3 evaluates the efficiency of the collision prediction component. Finally, Section 5.4 evaluates the FPGA resource utilization of the HW PD only and the complete system that includes the HW PD, HW SGM, and the HW/SW communication interface.

### 5.1. Location Evaluation

The database, evaluation strategy, and system parameters are presented to evaluate the pedestrian location. Then, results and analyses are conducted.

#### 5.1.1. Database

The database [33] was adopted for pedestrian location evaluation that provides the ground-truth bounding boxes and distances from the pedestrian to the vehicle in each frame for both training and testing samples. This database consists of 68 samples containing a sequence of stereo frames and the vehicle velocity and yaw rate. The image resolution is 1176 × 640 pixels, and the data capture rate is 16 FPS. The samples also contain scenarios involving moving and stopped vehicles.

#### 5.1.2. Evaluation Strategy

The location evaluation considers the lateral (X) and longitudinal (Z) position of the pedestrian obtained from the Geometric Filtering step of the PCP system shown in Figure 11. The strategy proposed in [34] is adopted in this work to compare system output with ground truth. This strategy specifies a localization tolerance, i.e., the maximum positional deviation that allows for counting a correct detection. The tolerance Z = 30% and X = 10% from [34] is adopted, which means that, for example, at a 10 m distance, localization errors of ±3 m and ±1 m in the longitudinal and lateral positions, respectively, are accepted as correct.

The test base is divided concerning the distance between the pedestrian and the vehicle. Group 1 is formed by the frames with distances between 7 and 25 m, while Group 2 contains frames between 7 and 50 m. There exist 2666 and 4305 frames for Groups 1 and 2, respectively.

#### 5.1.3. Systems Configuration Details

To demonstrate improvements when detecting both near and distant pedestrians, first the results from our previous work were reproduced [32]. Two detectors in high-level (HL) using OpenCV library functions were evaluated separately, one detector with windows of 64 × 128 (W64_H128_D3_HL) and another with windows of 48 × 96 (W48_H96_D3_HL). Next, these two detectors were combined (W64_H128_W48_96_D3_HL) and evaluated. The pyramid depth of all these detectors was set to 3 (i.e., $D_{\mathrm{pyr}}=3$).

Two SVM-Ps were defined to demonstrate similar results as in high-level. The modules ${\mathrm{SVM}-P}_{1}$ and ${\mathrm{SVM}-P}_{2}$ have detection windows of 64 × 128 and 48 × 96, respectively. ${\mathrm{SVM}-P}_{1}$ and ${\mathrm{SVM}-P}_{2}$ are responsible for detecting closer and further pedestrians, respectively. The accuracy of the proposed HW PD system with the two SVM-Ps combined was evaluated (i.e., $Q_{\mathrm{SVM}-P}=2$). To demonstrate the importance of the image pyramid, two detectors with different pyramid depths were evaluated, one detector having one level (W64_H128_W48_96_D1_HW) and another having three levels (W64_H128_W48_96_D3_HW). The fractional part of all the detectors is 8 bits wide (i.e., $F_{\mathrm{frac}}=8$).

To train each SVM-P and the high-level detectors for a given database, positive and negative patches are created from training samples following strategies similar to [6,34]. The ready-made function from the OpenCV library is used for SVM training. Data augmentation is applied for each positive crop using horizontal mirroring, image rotation, and contrast changing. A Bootstrapping algorithm also is applied to generate negative patches. These data augmentation parameters have been carefully defined to enable detector accuracy convergence during the training phase and are detailed in our previous work [6].

The following parameters are common to all evaluated systems. The dimensions of the HOG cell and block in pixels are, respectively, 8 × 8 and 16 × 16. The detection window stride in both directions is 8 pixels. The number of bins of the HOG histogram is set to 8. The pyramid scale parameter $S_{\mathrm{pyr}}$ is 1.1.

The parameters of the SGM module [24] are the penalties $P_{1}$ and $P_{2}$, which define how smooth the disparity data is, the valid disparity threshold $\sigma_{\mathrm{SGM}}$ which defines the sensitivity to detect a mismatched disparity, and the disparity number $N_{d}$ that defines the distance range supported. The parameter $P_{1}$, $P_{2}$, $N_{d}$, $\sigma_{\mathrm{SGM}}$ are set, respectively, to 24, 120, 128, and 20. The EKF parameters are process noise $\sigma_{x}$, measurement noise $\sigma_{u}$ and $\sigma_{d}$, and the initial covariance $P_{0}$. The parameters $\sigma_{x}$, $\sigma_{u}$, $\sigma_{d}$, and $P_{0}$ were defined, respectively, as 4.0, 6.15, 0.32, and 0.01. Details about all other PCP system parameters can be found in [6].

#### 5.1.4. Results and Analysis

**Figure 13:**
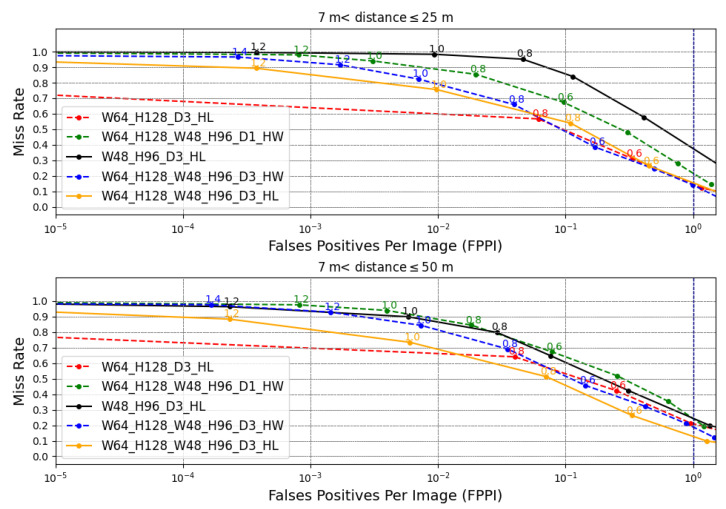
Quality results of some detectors when locating pedestrians, measured by FPPI and MR. The database is split into distance groups. Group 1 and Group 2 refer to pedestrians between 7 and 25 and between 7 and 50 m, respectively. $\sigma_{SVM}$ values are generated in the interval of [2.0,0.0] with steps of 0.2 to obtain these results. Each label near the point is the confidence value. The term D is a simplification to $D_{\mathrm{pyr}}$. HL means high-level, stating that the detection function used is from the OpenCV library.

The detectors are analyzed by miss rate versus false positive per image (FPPI) [35] as shown in Figure 13. The miss rate is the ratio of the number of pedestrians that were not detected by the total number of frames, and the FPPI is the ratio of the total number of false positives by the total number of frames. It is considered a false positive when the detector reports that there is a pedestrian when there is not.

It is essential to clarify that the miss rate (*y*-axis) and FPPI (*x*-axis) are detector results. The detector parameter to obtain these two results is the detector confidence $\sigma_{SVM}$ that has values in the discrete interval of [2.0,0.0] with steps of 0.2. For each confidence value, the miss rate and FPPI are obtained. Each label near to the point are confidence values in Figure 13. The higher confidence value indicates that the detector is more accurate in affirming that a window contains pedestrians, but fewer pedestrians are detected and, consequently, a high miss rate. On the other hand, higher FPPI is permitted with lower confidence, so the miss rate decreases, since more false positives per image are accepted, and therefore, true positives have a higher chance of being detected. It is desired to have detectors with a low value of miss rate and a low value of FPPI. Typically, pedestrian detection works adopt 1 FPPI (i.e., $10^{0}$ FPPI) as a common reference point to compare detector results.

As shown in Figure 13, the detector W64_H128_D3_HL achieved, in Group 1, a better detection performance than the W48_H96_D3_HL, with a 14% miss rate against 38%. This result is due to the fact that, in this distance range, the pedestrian dimension fits better in the detector with window of 64 × 128. In Group 2, the combined detector W64_H128_W48_96_D3_HL achieved a better result with a missing rate of 12% in 1 FPPI. The combination made it possible to capture pedestrians better in these two ranges of distances.

In evaluating the HW PD systems, it is possible to note that the detector with pyramid level 3 resulted in better results than the detector of level 1 in both distance groups. For 1 FPPI, the detector W64_H128_W48_H96_D3_HW achieved a 13% miss rate in Group 1 against 21% from the detector W64_H128_W48_H96_D1_HW. In Group 2, the detector W64_H128_W48_H96_D3_HW achieved an 18% miss rate against 23% from the detector W64_H128_W48_H96_D1_HW. The improvement with the increase in the pyramid level occurs because the greater the depth, the greater the ability to locate larger, and therefore closer, pedestrians [24].

It is possible to note also a slight reduction in the quality of the proposed HW PD system compared to the HL PD system. The main reasons for this reduction are that the HL PD system processes floating-point data, its HOG approach has trilinear interpolation techniques that consider the bin spatiality, and Gaussian weighting that reduces the edge effect that impairs detection [20].

As can be seen, detectors tend to saturate the miss rate value. Detectors that saturate in a lower miss rate are more attractive. As shown in Figure 13, increasing the depth of the pyramid and increasing the number of windows of different dimensions allowed the reduction of this miss rate. A strategy for defining the best distance range for each SVM-P could be developed to improve the accuracy of the entire system. Other ways to improve the miss rate that can be done from the proposed detector is to reduce scan stride and join the HOG with other feature extractors. The first solution increases the image coverage and, consequently, the chance of finding more pedestrians. The second increases the number of salient pedestrian features to improve classification ability [35]. Local binary patterns (LBP) [36], stereo disparity features [34], and convolutional neural networks (CNN) [37] are examples of feature extractors.

### 5.2. Processing Performance Evaluation

The processing performance of the proposed PD system and the PCP system using the HARPv2 platform were estimated. The performance results are in terms of latency and frames per second (FPS), estimated through an average of 1000 frames processed with outliers removed due to operating system scheduling.

**Figure 14:**
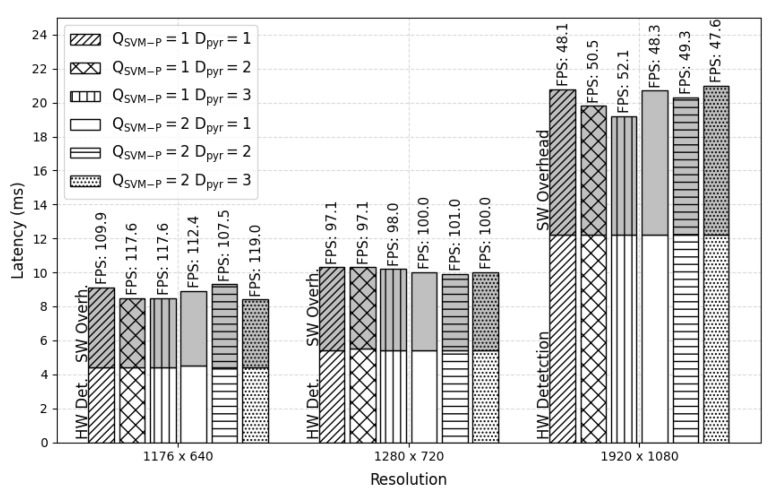
Processing performance of our HW PD system in the HARPv2 platform. The terms Det and Overh mean detection and overhead, respectively.

Figure 14 shows detector results at various image resolutions, pyramid depth ($D_{\mathrm{pyr}}$), and the number of SVM-P ($Q_{\mathrm{SVM}-P}$). The time spent preparing the image in shared memory and assembling the bounding boxes (SW Overhead) and the hardware detection processing time (HW Detection) was estimated and presented in each vertical bar.

A significant result noted in Figure 14 concerns the time to perform detection processing in hardware, which remains approximately constant in all tested configurations for a given resolution. If the pyramid depth and the number of SVM-Ps are further increased, the system will not reduce the processing performance. It is possible because all the resizes, HOGs, and SVMs modules are processed parallel and without frame loss.

Table 1 shows PCP system results at various image resolutions. Since the performance is constant concerning $D_{\mathrm{pyr}}$ and $Q_{\mathrm{SVM}-P}$, these parameters were set to 3 and 2, respectively. An important result is that the PD processing response latency and SGM are approximately similar to the PD module. The reason is that the two modules run in parallel and deliver the results almost simultaneously. Furthermore, the processing time of the two modules is quite short compared to the latency of the entire PCP system (Whole System). The biggest time bottleneck is in the communication functions between hardware and software. Any improvement in these functions or even reducing the number of data transmitted will result in the PCP system processing performance gains.

### 5.3. Collision Prediction Evaluation

The effectiveness of the PCP system on the collision prediction was evaluated with the improvement of the PD system processing performance in hardware. This evaluation defines the database used, evaluation strategy, and the configuration of the systems being assessed, and presents results and analyses.

#### 5.3.1. Database

**Figure 15:**
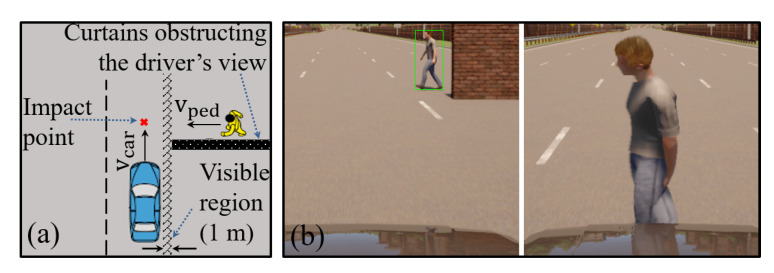
Evaluation scenario from [38]: (**a**) bird’s-eye view (**b**) Screenshots from the CARLA.

Since it was not possible to find a collision database, an evaluation database was built from a kind of scenario defined on [38], as shown in Figure 15a. This scenario involves a pedestrian moving perpendicularly towards the vehicle and occluded by a wall. The car strikes the pedestrian at approximately 50% of the vehicle’s width without any braking action. The database building strategy is similar to our work [6] using the Cars Learning to Act (CARLA) simulator version 0.9.7 [39].

The parameters for the scenarios created are the vehicle velocity $\left(V_{\mathrm{car}}\right)$ and the time-to-collision (TTC). The TTC parameter (in seconds) is the quotient of the vehicle distance to pedestrian (in meters) to its speed (in m/s) at the time of the appearance of crash risk [38].

Following [38], the values for $\left(V_{\mathrm{car}}\right)$ are 20, 30, 40, 50, and 60 km/h, and the TTC values are 0.6, 1.0, 1.4, 1.8, 2.2, 2.6, and 3.0. The sampling rate of the frames is 120 FPS, and the resolution of the frames is 1280 × 720 pixels. The pedestrian position and movement parameters were carefully adjusted to ensure that the collision time matches the TTC of each scenario when the pedestrian becomes visible.

All the combinations between $V_{\mathrm{car}}$ and TTC were performed, generating 35 scenarios for a type of pedestrian. These combinations were repeated for two more different pedestrians. In total, 35×3=105 test scenarios were created. The training base was created without involving collisions. Four types of pedestrians different from the test base were introduced, vehicles and pedestrians moving and stopped, and four CARLA environments. In total, 100 training scenarios were built. Each frame’s pedestrian bounding box, vehicle’s velocity, and yaw rate were annotated. Some screenshots of the CARLA scenario are presented in Figure 15b.

#### 5.3.2. Evaluation Strategy

The PCP system’s efficiency is assessed by checking the distance at which the system predicted a collision for the first time from the moment the pedestrian appeared (the first collision prediction). The safe distance supports a prediction assessment that ensures that the vehicle will not collide with the pedestrian if the system predicts the collision above that distance. This safe distance [40] is defined as:

(18) $$\begin{array}{c} dist_{\mathrm{safe}}\left(V_{\mathrm{car}},a_{b},T_{r}\right)=\frac{V_{\mathrm{car}}^{2}}{2\cdot a_{b}}+T_{r}\cdot V_{\mathrm{car}}\;\;\;\left(\mathrm{meters}\right), \end{array}$$

where $a_{b}$ is the maximum deceleration of the vehicle measured in m/s${}^{2}$, and $T_{r}$ is the driver’s reaction time to press the brake pedal measured in seconds. The average driver reaction time is around 1.0 s, and average deceleration is around −4.5 m/s${}^{2}$[38]. These values were used for $T_{r}$ and $a_{b}$.

#### 5.3.3. Systems Configuration Details

The HW PD system (W64_H128_W48_96_D3_HW) was evaluated within the PCP system (now it is called PCP/HW-PD). In this same PCP system, the HL PD system (W64_H128_W48_96_D3_HL) was also assessed (now it is called PCP/HL-PD). The YOLOv3-based PD system was also evaluated (PCP/YOLOv3). HL-PD-based and YOLOv3- based PD systems run on RTX 2070 GPU with 8GB of memory. The processing rates of the PCP/HW-PD, PCP/HL-PD, and PCP/YOLOv3 systems in the synthetic database are 60 FPS, 30 FPS, and 12 FPS, respectively. Since the database sampling rate is 120 FPS, capture intervals were defined that correspond to the actual rates of each PCP system. The intervals for the PCP/HW-PD, PCP/HL-PD, and PCP/YOLOv3 are 2, 4, and 10 frames, respectively.

The PCP system parameters are the same as defined in Section 5.1.3. The difference is in the process noise parameter $\sigma_{x}$ of the EKF filter. The optimization method adopted is defined in [33] with test samples from the synthetic database used to find the best value of $\sigma_{x}$ for each PCP system. For PCP/HW-PD, PCP/HL-PD, and PCP/YOLOv3, the values are 20.1, 60.5, and 80.3, respectively.

The training strategy is the same as described in Section 5.1.3. The synthetic training samples are used to train each SVM-P. For training YOLOv3, the author’s methods [41] are followed that use full images with no negative sample added from bootstrapping. The same full images used to train the proposed detector are employed to train the YOLOv3. The Darknet neural network framework for training and testing [41] is used that performs multi-scale training, lots of data augmentation, batch normalization, and all of the standard stuff.

#### 5.3.4. Results and Analysis

**Figure 16:**
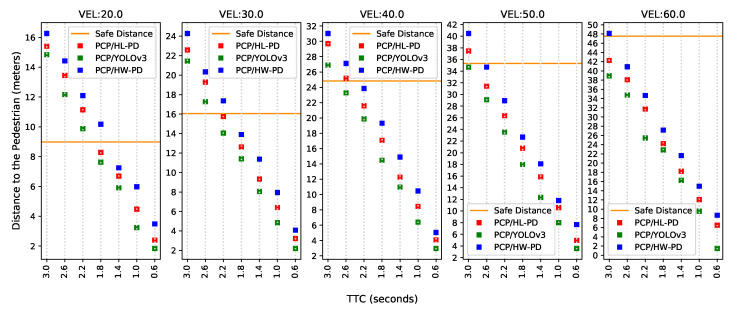
First collision prediction since the pedestrian’s emergence. The comparison was made involving our PCP system (PCP/HW-PD), PCP system with OpenCV-based detector (PCP/HL-PD) and PCP system with YOLOv3-based detector (PCP/YOLOv3).

It can be noted in Figure 16 that the proposed HW-PD system can predict more collisions at a safe distance than the other PDs. For example, 11 safe predictions were counted with HW-PD system against 8 using the OpenCV (PCP/HL-PD) and 6 using YOLOv3 (PCP/YOLOv3). The main reason for this is that once the processing time is longer, estimating the correct pedestrian speed through the EKF will also take longer.

**Figure 17:**
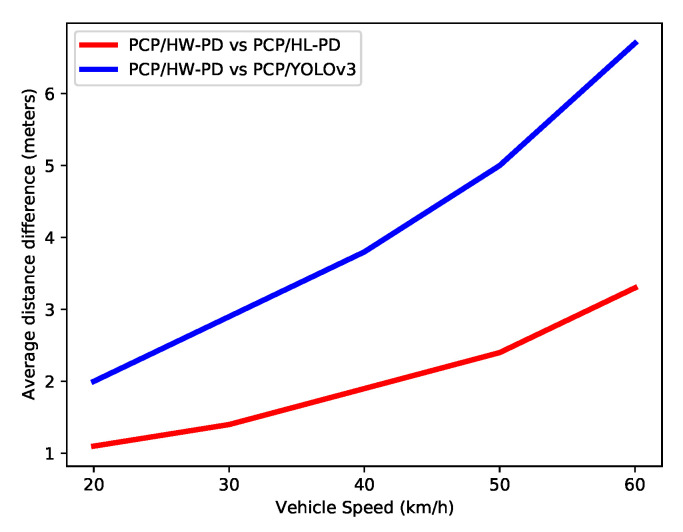
Average distance difference in meters for decision-making calculated from the results of all TTCs for each speed.

Although it is not always possible to guarantee safe distance, it is possible to note an increase in decision-making distance with the HW PD system (PCP/HW-PD) at all speeds and TTC configurations. To better evidence this distance gain, the average, at each velocity, of the distance differences between the proposed system and two others (i.e., PCP/HL-PD and PCP/YOLOv3) for all TTC values were calculated, as shown in Figure 17.

It is possible to observe in Figure 17 that the techniques and processing rate of the proposed system resulted in an increase in the distance for decision-making in comparison with other systems. For example, compared with YOLOv3, our system can detect a collision 6 m in advance for a car speed of 60 km/h. This increase can mitigate the impact and severity of the crash.

This difference increases almost linearly with the vehicle speed. This is due to the response time of the proposed detection system, which is shorter than other systems. Since several frames are needed for collision estimation, this time difference leads to an increased gain in the distance with faster vehicles. This linear growth brings more safety to the pedestrian, especially in cars at higher speeds. Efforts to reduce the response time of the PCP system will increase the safety distance even further. Specifically, in the PCP system proposed in this work, solving the communication bottlenecks due to the platform, as mentioned in Section 5.2, means that this system will have a shorter response time.

### 5.4. FPGA Resources Evaluation

Table 2 presents FPGA synthesis data of the PD module on the Arria 10 platform obtained for different values of image resolutions, SVM-Ps quantity, and pyramid depth as presented. In addition, synthesis data were obtained from the whole hardware system involving the PD, SGM, and communication modules as shown in Table 3.

As may be observed in Table 2 the increase in the image resolution almost does not impact the number of memory blocks, amount of LUTs, and registers. This feature is due to the reusability of modules to process all image pixels. It is notable that the number of processing resources did not double when increasing the amount of SVM-P. This result is due to the reuse of the same image pyramid for both SVM-Ps.

### 5.5. Existing PD Systems

Table 4 presents the results of some hardware-based existing PD systems concerning hardware occupation, operational frequency, and the processing rate for some resolution settings and pyramid depth. The processing speed is evaluated by millions of detection windows per second (MWPS), which signifies the product of the number of detection windows scanned in a frame and the FPS.

The result in terms of MWPS of the proposed PD system was measured in the prototyping platform, considering the platform communication overhead. Our system has a higher MWPS than most of the works. Although [22] achieves better FPS results, the system works at a higher frequency (270 Mhz) compared to our system (150 Mhz). Resource occupancy comparison with [22] is not possible because of the FPGA platform difference. Furthermore, it is impossible to perform a comparison in terms of accuracy due to the lack of database compatibility. The INRIA database [20], used by most of these works, does not provide stereo frames data nor information on vehicle movement and geometry that is needed by our system.

## 6. Conclusions

This work proposed an FPGA-based implementation of the pedestrian detection (PD) system based on HOG and SVM techniques, supporting image pyramid and detection windows of different dimensions. It was demonstrated that with detection windows of various sizes, the PD system’s miss rate was reduced by at least 6% compared to another system processing detection windows of unique dimensions. Specific strategies for each step are proposed to ensure the throughput of one pixel per clock cycle. For the most critical SVM module, a novel approach was proposed to process many detection windows through units using parallel and pipeline processing to process a different set of windows. This approach provided a gain in processing performance compared with other works and a constant performance independent of the pyramid depth and quantity of different-size detection windows. The processing performance gain of the proposed PD system inside the PCP system managed to avoid more collisions compared to lower systems and ensured an improvement in decision-making distance of a maximum of 3.3 m. Future works include scalability improvements to the proposed PD system to process more windows of different dimensions and more pyramid levels.

## Figures and Tables

**Table 1:** Processing performance of the PCP System na plataforma HARPv2. The operating frequency of the detection and stereo matching modules in all configurations was 150 MHz.

Resolution	Performance			
**Resolution**	**Whole System ^1^**	**GPP Steps ^2^**	**Overh. ^3^**	**FPGA Steps ^4^**
1176 × 640	74.1 (13.5 ms)	0.1 ms	9.8 ms	3.8 ms
1280 × 720	66.2 (15.1 ms)	0.1 ms	10.3 ms	5.4 ms
1920 × 1080	28.2 (35.4 ms)	0.2 ms	24.6 ms	11.1 ms

^1^ Processing performance of the whole heterogeneous PCP system measured by FPS (latency in milliseconds). ^2^ Processing time of all GPP-based steps as described in Figure 12 measured in milliseconds. ^3^ Overhead in milliseconds to arrange image data in the shared memory and mount the GPP memory results to post-processing. ^4^ Processing time of the SGM and PD modules measured in milliseconds.

**Table 2:** Hardware resource of the PD system.

Image Resolution	Module Description	Module Parameters	Hardware Resource		
			KALMs ^3^	KReg. ^4^	RAM ^5^
1280 × 720	Resize	-	1.3 (0.3%)	3.3	0.21 (0.4%)
	Gradient	-	1.7 (0.4%)	2.4	0.12 (0.2%)
	Cell	-	1.0 (0.2%)	1.7	0.05 (0.1%)
	Block	-	1.7 (0.4%)	7.5	0.24 (0.4%)
	SVM	${N_{c}}^{1}\;=4$	12.3 (2.9%)	24.5	2.42 (4.4%)
	Whole ^2^	$D_{\mathrm{pyr}}=1$ $Q_{\mathrm{SVM}-P}=1$	16.9 (4.0%)	37.3	3.03 (5.5%)
	Whole	$D_{\mathrm{pyr}}=2$ $Q_{\mathrm{SVM}-P}=1$	34.8 (8.2%)	77.1	6.41 (11.7%)
	Whole	$D_{\mathrm{pyr}}=3$ $Q_{\mathrm{SVM}-P}=1$	52.5 (12.4%)	116.6	9.57 (17.3%)
	Whole	$D_{\mathrm{pyr}}=3$ $Q_{\mathrm{SVM}-P}=2$	79.7 (18.9%)	172.4	13.75 (24.9%)
1920 × 1080	Resize	-	1.3 (0.3%)	3.3	0.41 (0.8%)
	Gradient	-	1.7 (0.4%)	2.4	0.12 (0.2%)
	Cell	-	1.0 (0.2%)	1.7	0.05 (0.1%)
	Block	-	1.7 (0.4%)	7.5	0.24 (0.4%)
	SVM	${N_{c}}^{1}\;=4$	12.8 (3.0%)	24.8	2.73 (4.9%)
	Whole	$D_{\mathrm{pyr}}=1$ $Q_{\mathrm{SVM}-P}=1$	17.3 (4.0%)	37.5	3.26 (5.8%)
	Whole	$D_{\mathrm{pyr}}=2$ $Q_{\mathrm{SVM}-P}=1$	35.6 (8.4%)	78.1	6.85 (12.4%)
	Whole	$D_{\mathrm{pyr}}=3$ $Q_{\mathrm{SVM}-P}=1$	54.0 (12.7%)	118.7	10.45 (18.9%)
	Whole	$D_{\mathrm{pyr}}=3$ $Q_{\mathrm{SVM}-P}=2$	82.3 (19.4%)	176.2	14.78 (26.8%)

^1^ Amount of parallel processing columns of SVMs. ^2^ For the whole PD system, $N_{c}=4$. ^3^ Amount of adaptive logic modules (ALMs) in units of $10^{3}$. ^4^ Amount of registers in units of $10^{3}$. ^5^ Amount of RAM storage measured in megabits.

**Table 3:** Resource usage of the whole HW system that involves PD, SGM, and HW/SW communication.

Image Resolution	Module Description	Module Parameters	Hardware Resource		
			KALMs ^2^	KReg. ^3^	RAM ^4^
1280 × 720	Whole PD	$D_{\mathrm{pyr}}=3$ $Q_{\mathrm{SVM}-P}=2$	79.7 (18.8%)	172.4	13.7 (24.9%)
	SGM	${N_{d}}^{1}\;=128$	73.7 (17.4%)	96.2	5.5 (10.0%)
	HW/SW Comm. ^5^	-	41.8 (9.8%)	55.1	0.9 (1.6%)
	Whole System	$D_{\mathrm{pyr}}=3$ $Q_{\mathrm{SVM}-P}=2$ $N_{d}=128$	169.7 (40.1%)	343.2	24.3 (44.2%)
1920 × 1080	Whole PD	$D_{\mathrm{pyr}}=3$ $Q_{\mathrm{SVM}-P}=2$	82.3 (19.4%)	176.2	14.7 (26.7%)
	SGM	$N_{d}=128$	79.1 (18.7%)	106.9	8.8 (16.0%)
	HW/SW Comm.	-	41.8 (9.8%)	55.1	0.9 (1.6%)
	Whole System	$D_{\mathrm{pyr}}=3$ $Q_{\mathrm{SVM}-P}=2$ $N_{d}=128$	185.1 (43.7%)	361.9	28.6 (52.0%)

^1^ Disparity Range. ^2^ Amount of adaptive logic modules (ALMs) in units of $10^{3}$. ^3^ Amount of registers in units of $10^{3}$. ^4^ Amount of RAM storage measured in megabits. ^5^ Communication.

**Table 4:** Hardware occupation and the processing rate obtained from existing pedestrian detection systems.

Work	Device	Configuration	Hardware Resource				Performance		
			KLUTs ^2^	KReg. ^6^	RAM ^3^	Ext.RAM ^1^	MHz	FPS	MWPS ^4^
[13] (HOG + SVM)	Zynq 7020	1920 × 1080, 1 level	11.5	12.2	$n_{u}$ ^1^	$n_{u}$	150	60	1.9
[19] (HOG + SVM)	Cyclone IV	800 × 600, 1 level	16.0	7.2	0.3	$n_{u}$	150	162	1.2
[42] (Pyramid + HOG + SVM)	Cyclone IV	1920 × 1080, 9 levels	47.2	25.1	4.2	$n_{u}$	140	33	9.5
[43] ( HOG + SVM )	Cyclone V	640 × 480, 1 level	8.4	$n_{i}$ ^1^	0.2	$n_{u}$	84	273	1.3
[22] ( Pyramid + HOG + SVM )	Virtex-5	1920 × 1080, 6 levels	38.5	43.0	7.1	$n_{u}$	270	64	10.0
[14] ( YOLO )	Virtex-7	416 × 416	155.0	$n_{i}$	21.9	$n_{u}$	200	60.1	-
Ours ( Pyramid + Multi-Windows ^5^ + HOG + SVM )	Arria-10	1920 × 1080, 6 levels	82.3	176.2	14.8	$n_{u}$	150	52.1	10.0

^1^ The terms $n_{i}$, $n_{u}$, and Ext.RAM, respectively, signify not informed, not used, and external memory. ^2^ $10^{3}$ LUTs. ^3^ Amount of RAM storage measured in megabits. ^4^ Million of detection windows per second. ^5^ Detection windows with different sizes. ^6^ Amount of registers in units of $10^{3}$.
